# Preparation, Characterization, and Evaluation of Pyraclostrobin Nanocapsules by In Situ Polymerization

**DOI:** 10.3390/nano12030549

**Published:** 2022-02-05

**Authors:** Bingna Huang, Feifei Chen, Yue Shen, Changcheng An, Ningjun Li, Jiajun Jiang, Chong Wang, Changjiao Sun, Xiang Zhao, Bo Cui, Zhanghua Zeng, Haixin Cui, Yan Wang

**Affiliations:** Institute of Environment and Sustainable Development in Agriculture, Chinese Academy of Agricultural Sciences, Beijing 100081, China; bingnahuang@163.com (B.H.); 18301262687@163.com (F.C.); shenyue@caas.cn (Y.S.); 13277762325@163.com (C.A.); liningjun09@126.com (N.L.); jiangjiajun05@163.com (J.J.); wangchong01@caas.cn (C.W.); sunchangjiao@caas.cn (C.S.); zhaoxiang@caas.cn (X.Z.); cuibo@caas.cn (B.C.); zengzhanghua@caas.cn (Z.Z.)

**Keywords:** pyraclostrobin, nanotechnology, pesticide nanocapsules, in situ polymerization

## Abstract

In this study, pyraclostrobin nanocapsules were prepared by in situ polymerization with urea–formaldehyde resin as a wall material. The effects of different emulsifiers, emulsifier concentrations, and solvents on the physicochemical properties of pyraclostrobin nanocapsules were investigated. Solvesso™ 100 was selected as the solvent, and Emulsifier 600# was used as the emulsifier, which accounted for 5% of the aqueous phase system, to prepare pyraclostrobin nanocapsules with excellent physical and chemical properties. The particle size, ζ potential, and morphology of the nanocapsules were characterized by a particle size analyzer and transmission electron microscope. The nanocapsules were analyzed by Fourier-transform infrared spectroscopy, and the loading content and sustained release properties of the nanocapsules were measured. The results show that the size of the prepared nanocapsules was 261.87 nm, and the polydispersity index (PDI) was 0.12, presenting a uniform spherical appearance. The loading content of the pyraclostrobin nanocapsules was 14.3%, and their cumulative release rate was 70.99% at 250 h, providing better efficacy and sustainability compared with the pyraclostrobin commercial formulation.

## 1. Introduction

Pesticides are necessary for preventing major biological disasters, protecting agricultural production, and ensuring the safety of national food production [1]. Pesticides technicial require processing into a variety of formulations before a pesticide can be put into practical production and applied in the environment. The characteristics of pesticide formulations, control objects and application methods determine the different application concentrations. At present, there are more than 50 pesticide formulations on the market, which can be roughly divided into two categories. One category is represented by traditional pesticide formulations, such as emulsifiable concentrates (ECs), wettable powders (WPs), and granules (GRs). The other encompasses green pesticide formulations, such as water emulsions (EWs), water-suspending agents (SCs), microemulsions (MEs), suspension emulsions (SEs), and water-dispersible granules (WDGs) [2]. However, some pesticide formulations have limitations such as a large particle size, low effective utilization rate, poor dispersion, and inconvenient transportation for practical applications [3,4,5]. Studies in recent years have shown that 90% of pesticides are not used effectively in the application process, posing a threat to the environment, and increasing application costs for farmers [6]. Therefore, the preparation of novel pesticide formulations using new materials and new methods has become necessary to improve pesticide performance, which is of great significance in agricultural production.

Nanotechnology is focusing on the study of properties and applications of materials at the nanoscale [7]. When the particle size reaches the nanometer level, particles are subject to the quantum size effect, small size effect, surface effect, and macroscopic quantum tunneling effect [8]. Throughout its development, nanotechnology has been gradually applied in many fields such as industry, military, medicine, and energy [9]. In agriculture, pesticide nanocapsule technology has recently been widely studied due to its good stability and sustained release property [10]. The use of nanotechnology to design and prepare targeted pesticides with environmentally responsive controlled release, via compound and chemical modifications, has also shown great potential in creating novel formulations [11]. Nanomaterials have the advantages of a small size, a large specific surface area, a strong adsorption capacity, and novel physical, chemical, and mechanical properties [12,13]. Their application in pesticide loading can significantly improve the stability of pesticides and help to improve the solubility and permeability of pesticides in target tissues [14,15]. Nanomaterials encapsulated pesticides: (a) As the particle size decreases, the specific surface area increases, thus increasing the affinity for the target, which reduces the amount of pesticide used. Moreover, nanomaterial envelopment of pesticides reduces the direct contact between pesticides and humans, which is a safe and environmentally friendly strategy [6,16,17]. (b) With decreasing capsule size, the adhesion and permeability of pesticides on the target are improved due to the small size effect and reduced likelihood of detachment, which reduces the loss of pesticides during use. (c) The dispersibility and wettability of nanocapsules are also better than those of conventional water-dispersible granules, and the cumulative release rate of pesticides increases with the reduction in capsule size, thus improving the efficacy of pesticides [18].

Pyraclostrobin is a fungicide discovered and developed by BASF in 1993, which is currently the most widely used methoxyacrylate fungicide [19,20]. Pyraclostrobin can effectively prevent the growth and spread of pathogenic bacteria, so it possesses not only a good bactericidal effect, but also a protective and therapeutic effect [21]. Pyraclostrobin also has wide application prospects in the pesticide preparation field due to its safety and environmental friendliness [22]. At present, the main formulations of pyraclostrobin include: GRs, WDGs, WPs, and SEs. In recent years, researchers have made some progress in the study of pyraclostrobin microcapsules or microspheres; however, most of the reported pyraclostrobin microcapsules or microspheres are still micron-scale capsules. The production of nanoscale capsules is always a hot topic in green pesticide formulations research in recent years in order to reduce the particle size of pyrostrobin, improve its efficiency, and reduce costs [23].

Urea–formaldehyde resin is an aqueous polymer formed by urea and formaldehyde catalyzed by an acid or alkali [24,25], which is widely used in agriculture as a coating material for slow-release pesticides with wide sources of raw materials, a low cost, a simple production process, stable performance, and ease of use [26,27,28]. Li et al. prepared a microencapsulated suspension of chlorpyrifos with urea–formaldehyde resin as the encapsulation, which had a good encapsulation effect [29]. In this study, pyraclostrobin nanocapsules were prepared by in situ polymerization with urea–formaldehyde resin as a capsule wall material, and the influence of different solvents and emulsifiers was explored in terms of particle size, dispersion, and retention efficiency, to provide a technical reference for the development of pyraclostrobin nanocapsules.

## 2. Materials and Methods

### 2.1. Materials

Pyraclostrobin (97%) and alkylphenol formaldehyde resin polyoxyethylene ether (emulsifier 700#) were purchased from Jinyue Biological Pharmaceutical Co., Ltd. (Beijing, China). Xylene, Sorbitan monooleate (Span 80), gelatin, Arabic gum, and sodium hydroxide (NaOH) were obtained from National Medicine Group Chemical Reagent Co., Ltd. (Beijing, China). Urea, dilute hydrochloric acid (HCl), and Glycerin were obtained from Beijing Chemical Factory (China). Formaldehyde was obtained from Baishi Chemical Co., Ltd. (Tianjin, China). Polyoxyethylene sorbitan monooleate (Tween 80) was purchased from Xilong Chemical Co., Ltd. (Shantou, China). Sodium dodecyl sulfate (SDS) was obtained from Fuchen Chemical Reagent Factory (Tianjin, China). Octylphenol polyoxyethylene ether-10 (OP-10) was sourced from Aladdin Biological Technology Co., Ltd. (Shanghai, China). Polyoxyethylene castor oil ether (EL-40) and dispersing agent NNO (NNO) were purchased from Yuanye Biology Co., Ltd. (Shanghai, China). Styryl phenol polyoxyethylene ether (emulsifier 600#) was purchased from Macklin Biochemical Technology Co., Ltd. (Shanghai, China). Sodium dodecyl benzene sulfonate (SDBS) was purchased from Yuanye Biological Technology Co., Ltd. (Shanghai, China). Solvesso™ 100 was obtained from Huishuo Technology Co., Ltd. (Shanghai, China). Methanol (high-performance liquid chromatography (HPLC) grade) was purchased from Fisher (Shanghai, China). Acetone was purchased from Gaojiaoyan Technology Co., Ltd. (Beijing, China). Pyraclostrobin suspension was sourced from BASF (Ludwigshafen, Germany). The water used in all experiments was purified using a Milli-Q water purification system (Merck, Darmstadt, Germany).

### 2.2. Methods

#### 2.2.1. Preparation of the Pyraclostrobin Nanocapsules

Pyraclostrobin nanocapsules were prepared by in situ polymerization using urea–formaldehyde resin as a wall material. Urea and formaldehyde were mixed with an emulsifier and deionized water (the molar ratio of urea to formaldehyde was 1:1.75), after which the system was adjusted to pH 8 in batches with 2% NaOH in a 30 °C water bath. Then, the temperature was gradually raised to 70 °C, the polycondensation reaction of urea and formaldehyde was initiated to form a urea–formaldehyde resin prepolymer, and an aqueous solution was obtained.

Pyraclostrobin was dissolved in a solvent to form an oil phase (mass ratio of pyraclostrobin to solvent was 1:3.65). The oil phase was then added dropwise to the aqueous phase and sheared to form a stable oil/water (O/W) emulsion. The emulsion was acidified by adjusting the pH of the system to 2.5 in batches with 2% dilute HCl acid in a 30 °C water bath. The curing reaction occurred for 1 h at 60 °C. Next, antifreeze glycerol and the suspension dispersant NNO were added. The solution was stirred and adjusted to pH 7 with 2% NaOH aqueous solution. A pyraclostrobin nanocapsule suspension was obtained. After centrifugation, washing three times with deionized water, and lyophilization, the pyraclostrobin nanocapsule lyophilized powder was prepared.

#### 2.2.2. Preparation of the Pyraclostrobin Nanocapsules Using Different Emulsifiers

Tween 80, OP-10, EL-40, Emulsifier 600#, Emulsifier 700#, Span80, SDS, SDBS, gelatin, and gum arabic were selected as emulsifiers in the nanocapsule system, with xylene as the solvent. The average particle size, 90% diameter percentile (D90), and dispersity (PDI) of the nanocapsules were determined, with the content of each emulsifier accounting for 2% of the aqueous system, in order to identify the optimal emulsifier.

#### 2.2.3. Preparation of the Pyraclostrobin Nanocapsules Using Different Solvents

Based on the types of emulsifiers explored in the xylene nanocapsule system, Change the solvent to Solvesso 100, which is similar to xylene but more environmentally friendly. Xylene and Solvesso 100 were used separately as the solvent, and the emulsifier contents of 1%, 2%, 3%, 5%, 7%, and 10% in the aqueous phase system were set to prepare pyraclostrobin nanocapsules and compare their properties.

#### 2.2.4. Characterization of Morphology and Particle sizes of the Pyraclostrobin Nanocapsules

The average particle size, PDI, and ζ potential of the samples were determined by dynamic light scattering (DLS) using a Zetasizer Nano ZS 90 (Malvern, Worcestershire, UK). Particle size was expressed as the mean size and D90. Each sample was measured in triplicate. Nanocapsule morphology was observed by a transmission electron microscope (TEM, Hitachi, Tokyo, Japan) with an acceleration voltage of 80 kV. The nanocapsules were analyzed by Fourier-transform infrared spectroscopy using the KBr method.

#### 2.2.5. Loading Content Determination of the Pyraclostrobin Nanocapsules

The loading content (LC) of the pyraclostrobin nanocapsules was determined at a wavelength of 295 nm by high-performance liquid chromatography (HPLC). An amount of 20 mg of pyraclostrobin nanocapsules was fully dissolved in 10 mL of acetone solution and dried via reduced-pressure distillation. Then, 50 mL of methanol was added to the dried pyraclostrobin nanocapsules for immersion, and the immersion solution was passed through the membrane filter to collect the filtrate for analysis. The loading content of the pyraclostrobin nanocapsules was calculated according to Equation (1).

LC (%) = (effective mass of pyraclostrobin/total mass of nanocapsules) × 100(1)

#### 2.2.6. Investigation of In Vitro Release Behaviors of the Pyraclostrobin Nanocapsules

To study the in vitro release behaviors of pyraclostrobin nanocapsules, 20 mg of pyraclostrobin nanocapsules was suspended in a dialysis bag with 5 mL of methanol/water mixture (70:30, *v/v*). Then, the dialysis bag was placed into a wide-mouth flask with a 95 mL mixture of methanol/water and incubated in an incubator shaker. Next, 5 mL of the mixed solvent outside the dialysis bag was withdrawn at various time intervals and replaced with a freshly mixed solution. The concentration of pyraclostrobin nanocapsules was measured using a UV–Vis spectrophotometer at a wavelength of 295 nm to determine the kinetic profile of the released pyraclostrobin nanocapsules. Technical pyraclostrobin and a commercial pyraclostrobin suspension were used as controls.

## 3. Results and Discussion

### 3.1. Preparation of the Pyraclostrobin Nanocapsules

**Figure 1:**
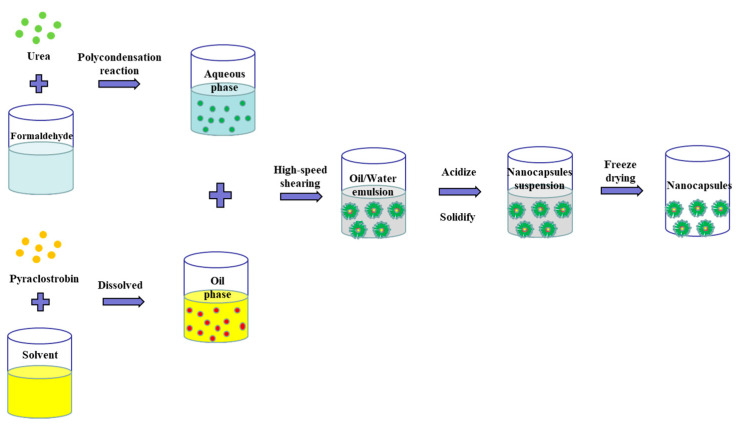
Schematic illustration of pyraclostrobin nanocapsules.

In this study, we prepared pyraclostrobin nanocapsules by in situ polymerization (Figure 1). Which refers to the in situ formation of one or several reinforcing phases or specific parts of the matrix through a chemical reaction under certain conditions [30,31]. We mixed the water phase with the oil phase, and prepared pyraclostrobin nanocapsules through the processes of high-speed shearing, acidification, solidification and freeze-drying.

### 3.2. Effect of Emulsifier Types on the Particle Size and Dispersibility of the Pyraclostrobin Nanocapsules

**Figure 2:**
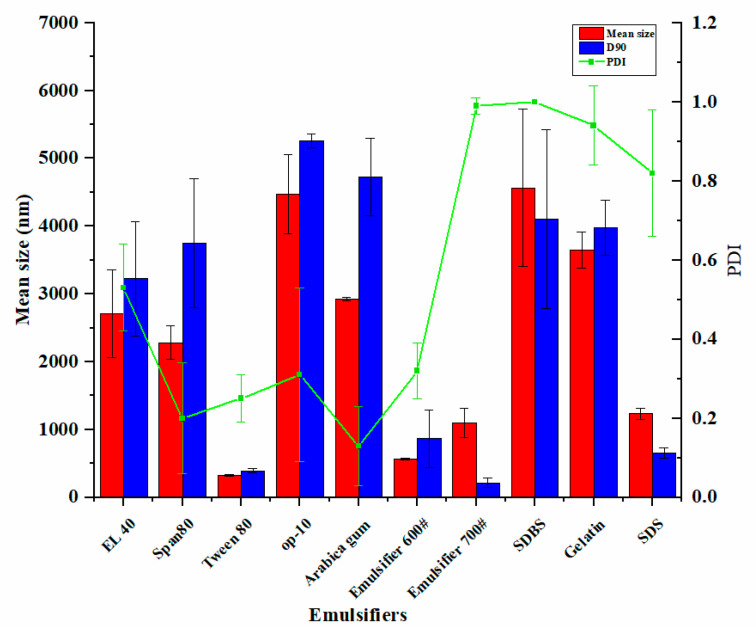
Effects of different emulsifiers on the particle size and PDI of nanocapsules.

We investigated the effect of the emulsifier types on the particle size and dispersibility of pyraclostrobin nanocapsules, including cationic, anionic, and non-ionic surfactants. Most emulsifiers have not reached satisfactory emulsifying effect for pyraclostrobin tants, (Figure 2). When Emulsifier 600# and Tween 80 were used as emulsifiers, the mean sizes of pyraclostrobin nanocapsules were less than 600 nm, which is conducive to the distribution and bactericidal activity of pesticides on crop leaves. In addition, the mean sizes of the particles prepared with Emulsifier 600# and Tween 80 were 326.70 nm and 569.83 nm, respectively. And the respective PDI values were 0.25 and 0.32. Tween 80 and emulsifier 600# are both hydrophilic surfactants, used as O/W emulsifiers in the preparation of pesticide microcapsules, and have good emulsification effects resulting in stable and uniform capsules. For these reasons, Emulsifier 600# and Tween 80 were chosen for further investigation.

### 3.3. Effect of Emulsifier Concentrations on the Particle Size and Dispersibility of the Pyraclostrobin Nanocapsules

**Figure 3:**
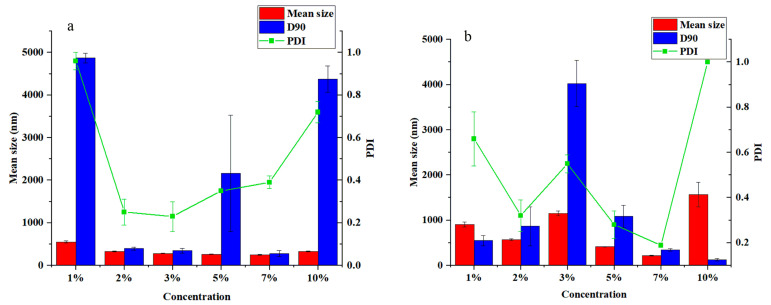
Effects of different concentrations of emulsifier on particle size and PDI of nanocapsules with xylene as solvent: (**a**) Tween 80, (**b**) Emulsifier 600#.

The concentration of the emulsifier also has an important influence on the particle size and dispersion of the system. Thus, pyraclostrobin nanocapsules were prepared with different concentration gradients of Tween 80 and Emulsifier 600#. The mean size and PDI results are shown in Figure 3. The mean size was 279.57 nm, and the PDI was 0.23, when Tween 80 reached an optimal concentration of 3% in the aqueous phase. The smallest average particle size was 216.53 nm, and the PDI was 0.19 when Emulsifier 600# was 7% of the aqueous phase. The mean size of nanocapsules prepared by other emulsifiers was greater than 300 nm, or the PDI was greater than 0.3. The results show that the average particle size, D90, and PDI of the nanocapsules prepared with the two emulsifier concentrations above were smaller, and the degree of dispersion was better. Under the optimal concentration, the average particle size, D90, and PDI of the nanocapsules prepared with 7% Emulsifier 600# were better than those prepared with 3% Tween 80 as the emulsifier.

### 3.4. Effect of Solvent on Morphology and Particle Size of the Pyraclostrobin Nanocapsules

**Figure 4:**
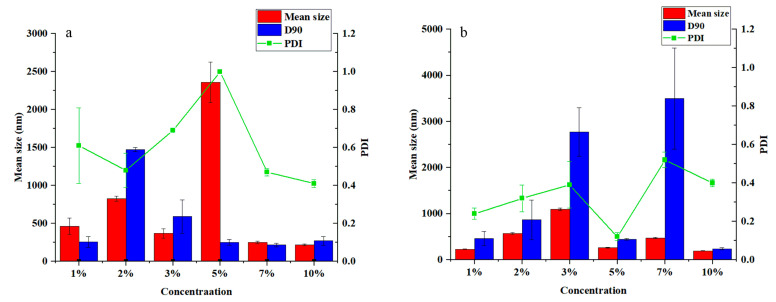
Effect of different emulsifier concentrations on nanocapsule size and PDI with Solvesso 100 as the solvent: (**a**) Tween 80, (**b**) Emulsifier 600#.

Because xylene is one of the most commonly used traditional solvents in the preparation of pesticides by in situ polymerization, we used xylene as the solvent in our previous research. However, xylene is toxic and unsafe to the operator. Therefore, the solvent was replaced with Solvesso 100, which has similar properties to xylene but is comparatively safer and more environmentally friendly. Under the same conditions of the preparation process for xylene, the emulsifier, emulsifier concentration, and nanocapsules were prepared separately. A group of preparation methods which resulted in an average particle size less than 300 nm and a PDI of less than 0.3 was selected to determine the optimal solvent, emulsifier, and concentration of emulsifier. The mean size and PDI results are shown in Figure 4. The dispersion system of Solvesso 100 was comparable to that of xylene when the dispersion of the obtained nanocapsules is better by Emulsifier 600# than that by Tween 80. the mean size of the optimal nanocapsules was 261.87 nm, and the PDI was 0.12 using Emulsifier 600# as the emulsifying agent with 5% concentration in the aqueous phase, which represents a relatively small particle size and better dispersion.

The zeta potentials of pyraclostrobin technical, empty capsules, and pyraclostrobin nanocapsules were measured and obtained as 5.19 ± 0.43 mV, −7.57 ± 1.11 mV, and −30.33 ± 0.76 mV, respectively. Based on the diffusion double layer theory, the urea–formaldehyde resin glue is negatively charged during its movement under alkaline conditions due to the glue core of the urea–formaldehyde resin glue which contains amino anions. Furthermore, layer with the high amount of -NH_2_, -OH, and -CH_2_-O-CH_2_- in the polymer structure of their adsorption, these hydrophilic groups can form a hydrated film on the surface of the adsorption layer, thus preventing the attack of cations in the diffusion layer. In this study, the high negative zeta potential values of the nanocapsules can be attributed to the negatively charged urea–formaldehyde resin capsule wall wrapped around the pyraclostrobin, indicating that the nanocapsules have good stability.

**Figure 5:**
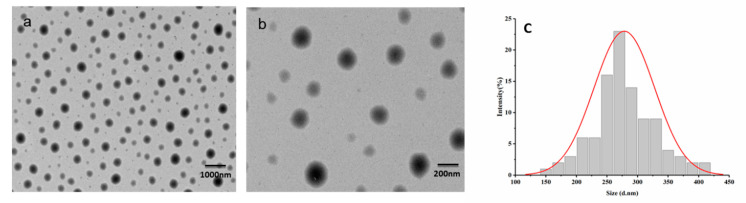
TEM image and particle size distribution illustration of the pyraclostrobin nanocapsules (using 5% Emulsifier 600# in the aqueous phase system, and Solvesso 100 as the solvent): (**a**) magnification of 3.0 k; (**b**) magnification of 15.0 k; (**c**) particle size distribution.

According to TEM images shown in Figure 5， the nanocapsules have a spherical structure at different magnification with the displayed particle size distribution.

**Figure 6:**
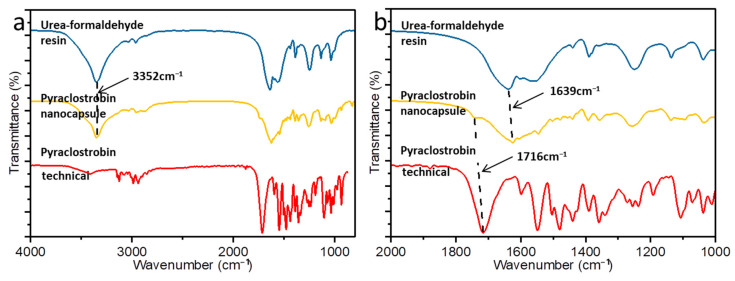
(**a**) Fourier-transform infrared spectra of urea–formaldehyde resin, pyraclostrobin nanocapsules, and technical pyraclostrobin. (**b**) Magnified regions of the spectra.

Fourier-transform infrared spectroscopy was performed to investigate pyraclostrobin encapsulation. Fourier-transform infrared spectrum of the nanocapsule shown in Figure 6 contained strong peaks at 1716 cm^−^^1^ corresponding to the stretching vibration of C=C on the benzene ring skeleton, which were attributed to pyraclostrobin. Peaks at 3352 and 1639 cm^−^^1^ corresponding to the amino N-H stretching vibration and the amide C=O vibration were attributed to the urea–formaldehyde resin. Other peaks characteristic of pyraclostrobin were not obvious, possibly because of strong absorbance by the urea–formaldehyde resin, which would have masked absorbance by the active ingredients. These spectra indicate that the urea–formaldehyde resin effectively encapsulated the pyraclostrobin in the nanocapsules.

### 3.5. Loading Content of the Pyraclostrobin Nanocapsules

The mean size and morphology of pyraclostrobin nanocapsules were improved by selecting types and concentrations of the above emulsifier and the solvent. With the change in emulsifier concentration, the particle size of nanocapsules changed as well, and the loading content of the pyraclostrobin nanocapsules also changed from 2.48% to 14.3%. This is because the urea–formaldehyde resin droplets with the smaller particle size were formed at relatively high emulsifier concentration, which increases the total surface area of urea–formaldehyde resin droplets as well as the mass of the capsule wall. Therefore, the loading content of nanocapsules increases with the increases with the higher emulsifier concentration. When the concentration of emulsifier is small, the pyraclostrobin is not emulsified sufficiently, and it is difficult for the urea–formaldehyde resin to wrap the pyraclostrobin, resulting in a low content of the nanocapsule. When the concentration of emulsifier is moderate, the urea–formaldehyde resin is emulsified into droplets with a small particle size, which increases the probability of an intact urea–formaldehyde resin coating on the pyraclostrobin, and thus the loading content of the nanocapsules is large. When the concentration of emulsifier is too high, the viscosity of the emulsion increases greatly, and the urea–formaldehyde resin has difficulty in coating the pyraclostrobin, meaning the loading content of the nanocapsule decreases. The loading content of pyraclostrobin nanocapsules was further evaluated under optimal preparation conditions, which involved 5% Emulsifier 600# in the aqueous phase system and Solvesso 100 used as the solvent. The loading content of the pyraclostrobin nanocapsules in the optimal system was determined by the method described in Section 2.2.5. The results show that the loading content of the pyraclostrobin nanocapsules was 14.3%. This indicates that the pyraclostrobin can be loaded effectively under the optimal conditions.

### 3.6. In Vitro Release Properties of the Pyraclostrobin Nanocapsules

**Figure 7:**
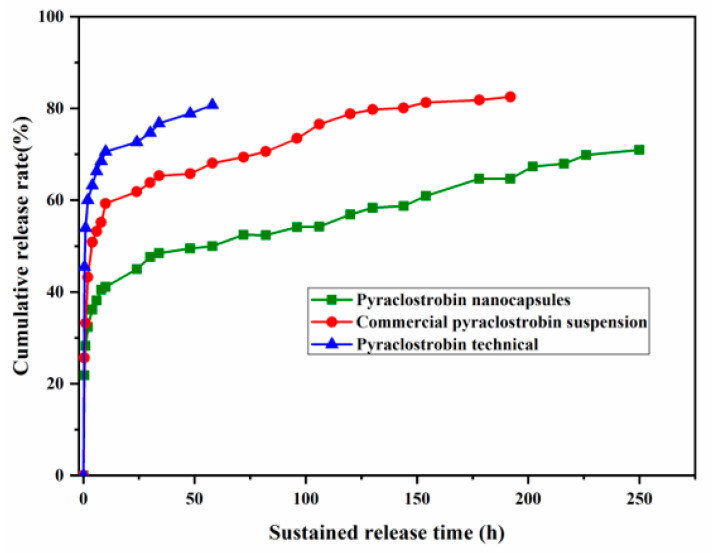
Cumulative release rates of pyraclostrobin nanocapsules, a commercial pyraclostrobin suspension, and technical pyraclostrobin.

Pesticides nanocapsules can improve the utilization rate of pesticides due to the encapsulation and controlled release function of nano-carriers. In this study, the method in Section 2.2.6 was used to determine the sustained release performance of the optimal system (using Solvesso 100 as the solvent and 5% Emulsifier 600# as the emulsifier). As shown in Figure 6, the cumulative release rate of the pyraclostrobin technical reached 80.76% at 58 h, the cumulative release rate of the commercial pyraclostrobin suspension reached 82.54% at 192 h, and the cumulative release rate of pyraclostrobin nanocapsules was 70.99% at 250 h (Figure 7). Compared with the pyraclostrobin technical and the commercial pyraclostrobin suspension, the pyraclostrobin nanocapsules had better sustainability. This indicates that the sustainable release of nanocapsules is beneficial to maintain longer efficacy, improve the utilization rate of pesticides, and reduce the spraying times and labor cost during the process of crop disease control in the field.

## 4. Conclusions

In this study, pyraclostrobin nanocapsules were successfully prepared using an in situ polymerization method. The effects of the emulsifier type, emulsifier concentration, and solvent of pyraclostrobin nanocapsules were investigated. The nanocapsules with the most optimal conditions were characterized by DLS, TEM, Fourier-transform infrared spectroscopy, zeta potential, and release curves. The results show that the pyraclostrobin nanocapsules have a smaller and more uniform size, better appearance characteristics, higher loading content, improved slow-release property and environmental friendliness. Therefore, this technology has a high potential to extend the effective duration and to reducing the use of pesticide, which helps to improve the utilization of pesticides.

## Data Availability

Data can be available upon request from the authors.

## References

[1] Wang C., Cui B., Zeng Z., Wang Y., Sun C., Zhao X., Feng L., Liu G., Cui H. (2017). Research Progress on Pesticide Solid Nanodispersion and Its Preparation Methods. J. Agric. Sci. Technol..

[2] Hu D. (2009). The Research on the Water Floating and Effervescent Dispersible Granules of the New Pesticide Formulation.

[3] Yu M., Sun C., Xue Y., Liu C., Qiu D., Cui B., Zhang Y., Cui H., Zeng Z. (2019). Tannic acid-based nanopesticides coating with highly improved foliage adhesion to enhance foliar retention. RSC Adv..

[4] Wang C., Cui B., Wang Y., Wang M., Zeng Z., Gao F., Sun C., Guo L., Zhao X., Cui H. (2021). Preparation and Size Control of Efficient and Safe Nanopesticides by Anodic Aluminum Oxide Templates-Assisted Method. Int. J. Mol. Sci..

[5] Cui B., Gao F., Zeng Z., Wang C., Wang Y., Sun C., Zhao X., Guo L., Shen Y., Liu G. (2020). Construction and characterization of avermectin B2 solid nanodispersion. Sci. Rep..

[6] Liu B., Wang Y., Yang F., Wang X., Shen H., Cui H., Wu D. (2016). Construction of a controlled-release delivery system for pesticides using biodegradable PLA-based microcapsules. Colloids Surf. B..

[7] Sun C., Wang Y., Zhao X., Cui B., Zeng Z., Cui H. (2018). Research on Preparation Technology of Polylactic Acid Nano-microsphere. J. Agric. Sci. Tech..

[8] Sun C., Cui H., Wang Y., Zeng Z., Zhao X., Cui B. (2018). Studies on Applications of Nanomaterial and Nanotechnology in Agriculture. J. Agric. Sci. Tech..

[9] Lin Z. (2010). Fundamentals and Applications of Nano Materials.

[10] Guo W., Cui R., Zhuang Z., Zuo W., Zhu Y., Gao J. (2017). Research Situation and Prospect of Pesticide Microcapsule. Mod. Agrochem..

[11] Huang B., Chen F., Yue S., Qian K., Wang Y., Sun C., Zhao X., Cui B., Gao F., Zeng Z. (2018). Advances in Targeted Pesticides with Environmentally Responsive Controlled Release by Nanotechnology. Nanomaterials.

[12] Ali I. (2012). New Generation Adsorbents for Water Treatment. Chem. Rev..

[13] Xu G., Wang J., Li C. (2012). Preparation of hierarchically nanofibrous membrane and its high adaptability in hexavalent chromium removal from water. Chem. Eng. J..

[14] Prasad R., Bhattacharyya A., Nguyen Q. (2017). Nanotechnology in Sustainable Agriculture: Recent Developments, Challenges, and Perspectives. Front. Microbiol..

[15] Camara M., Campos E., Monteiro A., Pereira A., Fraceto L. (2019). Development of stimuli-responsive nano-based pesticides: Emerging opportunities for agriculture. J. Nanobio-Technol..

[16] Cui B., Feng L., Pan Z., Yu M., Zeng Z., Sun C., Zhao X., Wang Y., Cui H. (2015). Evaluation of Stability and Biological Activity of Solid Nanodispersion of Lambda-Cyhalothrin. PLoS ONE.

[17] Yang D., Cui B., Zhao X., Zeng Z., Wang Y., Sun C., Liu G., Cui H. (2018). Comparative Study on Characterization and Field Efficacy Evaluation of New Pesticide Nanodispersion. J. Agric. Sci. Technol..

[18] Wang A., Wang Y., Sun C., Wang C., Cui B., Zhao X., Zeng Z., Yao J., Yang D., Liu G. (2018). Fabrication, Characterization, and Biological Activity of Avermectin Nano-delivery Systems with Different Particle Sizes. Nanoscale Res. Lett..

[19] Zuo W., Zhu Y., Zhuang Z., Cui W., Guo W., Liu Y., Fan J. (2017). Research Status and aparospects of Pyraclostrobin. World Pestic..

[20] Zhang Y. (2007). A novel methoxy acrylic acid vinegar bactericide-limonide vinegar. World Pestic..

[21] Hao H. (2015). Preparation and Characterization of the Pyraclostrobin Microcapsule Suspension Formulation.

[22] Yang L., Bai Y. (2012). Strobilurin Fungicide—Pyraclostrobin. Mod. Agrochem..

[23] Zhi Y., Wang G., Chen L. (2015). Preparation Technology and Development Situation of Pesticide Microcapsule. Synth. Mater Aging Appl..

[24] Zhang Y., Zhu L., Tan H. (2018). Adhesive and Bonding Technology.

[25] Yang B., Du F., Li Z. (2018). Adhesives—Formulation, Process and Equipment.

[26] Li J. (2021). Study on Gel, Morphology and Crystallization Characteristics of Urea-formaldehyde Resin.

[27] Wang H., Liu X., Jin C., Zhou Y., Ou X. (2017). Research Progress of Micro-capsule Suspension of Pesticide. Guangdong Chem. Ind..

[28] Gong S., Shen Z., Zhou X., Chen H., Xu H. (2018). Sustained-release Kinetics and Preparation Process Optimization of Chlorpyrifos/Urea-formaldehyde Resin Microcapsules. Mater. Rep..

[29] Li J., Dai L., Gu A., Bi Y. (2020). Preparation of 30% chlorpyrifos microcapsule suspension. JiangSu Agric. Sci..

[30] Larsen L. (2006). The Development of Wood Polymer Penetrant and In Situ Polymerization with Electron Beam and x-radiaton.

[31] Bergman R., Ibach R., Lapasha C., Denig J. (2009). Evaluating physical property changes for small-diameter, plantation-grown southern pine after in situ polymerization of an acrylic monomer. For. Prod. J..

